# Population pharmacokinetics of polymyxin B in critically ill patients with carbapenem-resistant organisms infections: insights from steady-state trough and peak plasma concentration

**DOI:** 10.3389/fphar.2025.1511088

**Published:** 2025-03-12

**Authors:** Jun Yang, Mingjie Yu, Yu Gan, Lin Cheng, Ge Yang, Lirong Xiong, Fang Liu, Yongchuan Chen

**Affiliations:** ^1^ Department of Pharmacy, The First Affiliated Hospital of Army Medical University, Chong Qing, China; ^2^ Department of Pharmacy, The First Affiliated Hospital of Chongqing Medical University, Chong Qing, China

**Keywords:** population pharmacokinetics, polymyxin B, critical illness, dosing optimization, consistency

## Abstract

**Aims:**

To establish a population pharmacokinetic (PopPK) model of polymyxin B (PMB) in critically ill patients based on steady-state trough (C_trough,ss_) and peak (C_peak,ss_) concentrations, optimize the dosing regimen, and evaluate the consistency of 24-hour steady-state area under the concentration-time curve (AUC_ss,24h_) estimation between model-based and the two-point (C_trough,ss_ and C_peak,ss_) methods.

**Methods:**

PopPK modeling was performed using NONMEM, Monte Carlo simulations were used to optimize PMB dosing regimens. Bland-Altman analysis was used to evaluate the consistency between the two AUC_ss,24h_ estimation methods.

**Results:**

A total of 95 patients, contributing 214 blood samples, were included and categorized into a modeling group (n = 80) and a validation group (n = 15). A one-compartment model was developed, with creatinine clearance (CrCL) and platelet count (PLT) identified as significant covariates influencing PK parameters. Simulation results indicated that when a Minimum Inhibitory Concentration (MIC) ≤ 0.5 mg·L^-1^, a probability of target attainment (PTA) ≥ 90% was achieved in all groups except for the 50 mg every 12 h (q12h) maintenance dose group. PTA decreased as CrCL increased, with slight variations observed across different PLT levels. The 75 mg and 100 mg q12h groups showed a higher proportion of AUC_ss,24h_ within the therapeutic window. Bland-Altman analysis revealed a mean bias of 12.98 mg·h·L^-1^ between the two AUC_ss,24h_ estimation methods. The Kappa test (κ = 0.51, P < 0.001) and McNemar’s test (P = 0.33) demonstrated moderate agreement, reflecting overall consistency with minor discrepancies in classification outcomes.

**Conclusion:**

The PopPK model of PMB is well-suited for critically ill patients. The 75 mg q12h and 100 mg q12h regimens are appropriate for critically ill patients, with CrCL levels guiding individualized dosing. A two-point sampling strategy can be used for routine therapeutic drug monitoring (TDM) of PMB.

## Introduction

The global spread of carbapenem-resistant organisms (CRO) has become a major public health concern, leading to increased morbidity and mortality due to their widespread resistance to common antibiotics (Tacconelli et al., 2018; Brink, 2019). Due to the lack of effective alternatives, polymyxin B (PMB) — an antibiotic that was initially withdrawn in the 1950s due to concerns over nephrotoxicity and neurotoxicity (Poirel et al., 2017) — has been reintroduced as a last-line agent against CRO infections (Abdallah et al., 2015; Rabanal and Cajal, 2017; Zhang et al., 2020). In China, where new antimicrobial options remain scarce, PMB now serves as a cornerstone therapy for these CRO. However, uncertainties persist regarding its optimal dosing, particularly due to limited pharmacokinetic (PK) data in CRO-infected populations and the dosing regimen continues to be debated (Bergen et al., 2010; Onufrak et al., 2017; Manchandani et al., 2018). PMB was often used in critically ill patients who present with distinct physiological traits, such as higher Acute Physiology and Chronic Health Evaluation II (APACHE II) scores, lower serum albumin levels, hemodynamic instability, and significant variability in creatinine clearance (CrCL) (Roberts et al., 2014a). These factors can alter the drug’s PK parameters, like clearance and volume of distribution, potentially leading to suboptimal antibiotic concentrations at the infection site (Roberts et al., 2014a). This suboptimal drug exposure is associated with bacterial tolerance and poor outcomes (Huemer et al., 2020), underscoring the need to optimize PMB dosing in this patient population.

Several studies have utilized population pharmacokinetic (PopPK) modeling to optimize PMB dosing in critically ill patients (Sandri et al., 2013a; Luo et al., 2022; Ye et al., 2022; Liang et al., 2023; Tang et al., 2023), Sandri et al. and Liang et al. conducted studies with 24 and 22 critically ill patients, respectively, both of which had relatively small sample sizes. Luo et al. included critically ill patients with and without continuous renal replacement therapy (CRRT), but their study did not fully capture the PK characteristics of patients without CRRT. Ye et al. focused on optimizing PMB dosing in critically ill patients with varying renal function, enrolling 23 patients. Tang et al. studied critically ill patients with nosocomial pneumonia, which limited the applicability of their findings to other types of infections. International guidelines and a clinical study from the Chinese population on PMB usage recommend a concentration-time curve at steady state over 24 h (AUC_ss,24h_) in the range of 50–100 mg·h·L^-1^ as the therapeutic window to ensure PMB safety and efficacy (Tsuji et al., 2019; Yang et al., 2022). The Chinese guidelines for therapeutic drug monitoring (TDM) of PMB (Liu et al., 2023) propose two methods for estimating the AUC_ss,24h_. One approach uses steady-state trough (C_trough,ss_) and peak (C_peak,ss_) concentrations with a first-order elimination equation, while the other employs individual PK parameters from a PopPK model to estimate AUC_ss,24h_. However, no studies have compared AUC_ss,24h_ estimates from these two methods, leaving the consistency of the results uncertain.

This study aims to: 1) to develop a PopPK model for PMB in critically ill patients with CRO infections to identify factors influencing PK variability in this population; 2) to select the optimal dosing regimen for this population based on Monte Carlo simulations of the final model; and 3) to assess the consistency between two AUC_ss,24h_ estimation methods, providing evidence for the TDM of PMB based on the two-point method using C_trough,ss_ and C_peak,ss_.

## Materials and methods

### Study design

A single-center prospective study was conducted in the intensive care unit (ICU) of the First Affiliated Hospital of Army Medical University from August 2021 to July 2024. Inclusion criteria: (1) patients aged ≥18 years; (2) patients receiving intravenous PMB for CRO infections confirmed by pathogen testing; (3) patients receiving at least four consecutive doses of intravenous PMB, or a loading dose followed by at least three consecutive doses. Exclusion criteria: (1) patients with missing clinical data; (2) patients hospitalized for fewer than 7 days; (3) patients receiving any form of renal replacement therapy during PMB treatment; (4) pregnant women. The research protocol was approved by the Ethics Committee of our hospital (No. (A) KY2021064).

### Data collection

Data collected from electronic medical records included: (1) demographic characteristics and main diseases; (2) PMB dose, administration route, and concurrent antibacterial agents; (3) routine blood test results; (4) liver and renal function indices, with CrCL calculated using the Cockcroft-Gault equation; (5) blood coagulation parameters; (6) other treatments such as extracorporeal membrane oxygenation (ECMO), mechanical ventilation, as well as relevant parameters such as the duration of these treatments.

### PMB sample collection and assay

After at least 48 h of treatment, two blood samples were collected: one immediately before the infusion and the other immediately after. The exact times of blood sampling and infusion were recorded for each patient. Plasma was separated by low-temperature, low-speed centrifugation and stored at −70°C until analysis.

PMB concentrations were analyzed using a validated ultra-performance liquid chromatography-tandem mass spectrometry (UPLC-MS/MS) method. Briefly, using polymyxin E2 as the internal standard, the assay was linear over 0.2–20.0 mg·L^-1^ and 0.05–5 mg·L^-1^ (r > 0.995) for PMB1 and PMB2 respectively. Intra-day and inter-day precision tests showed relative standard deviations (RSDs) ≤ 12.06%. The average extraction recovery ranged from 103.04% to 117.44%, and RSDs for the matrix effect and stability tests did not exceed 7.42%. PMB concentration was calculated as follows: total concentration of PMB = [PMB1 concentration/PMB1 molecular + PMB2 concentration/PMB2 molecular]*total PMB molecular (Liu et al., 2023).

### Population pharmacokinetic model

The PopPK model was developed using nonlinear mixed-effects modeling software: NONMEM (version 7.5.1, ICON plc, United States), Pirana (version 23.1.2, Certara L.P., United States), and PsN (version 5.3.0, https://uupharmacometrics.github.io/PsN/), with the first-order conditional estimation method including interaction (FOCE-I). The base model was selected based on goodness-of-fit (GOF) diagnostic plots, relative standard error (RSE) of parameters, and the objective function value (OFV). Spearman correlation was used to evaluate the relationships between covariates and individual empirical Bayesian estimates (EBEs) of PK parameters before covariate selection. Covariates included age, weight, APACHE II score, the presence of sepsis, and all laboratory parameters listed in Table 1. A decrease in OFV >3.84 (P < 0.05, χ^2^, df = 1) for forward addition and an increase >6.63 (P < 0.01, χ^2^, df = 1) for backward elimination were the criteria for covariate inclusion. GOF plots were used to assess the model. To assess the stability of the final model and the precision of the PK parameters, a bootstrap method with 1,000 resampling iterations was performed. The final model was then subjected to 1,000 simulations for visual predictive checks (VPC) to evaluate the model’s predictive ability and accuracy. The accuracy and predictive performance of the model were further assessed using normalized prediction distribution errors (NPDE) plots. Statistical validation of the model was conducted using the t-test, Fisher’s test, Shapiro-Wilk test, and the Global test.

**TABLE 1:** Demographic chacteristics and laboratory parameters for patients in PopPK model.

Characteristic	Modeling set	Validation set	P value
Age(y)	60 (47–74)	66 ± 5.26	0.84
Sex			
Male	60 (75%)	12 (80%)	
Female	20 (25%)	3 (20%)	
Total body weight (kg)	63 (55–74)	69.54 ± 3.25	0.93
Main diseases			
Severe infectious diseases	40 (50%)	7 (46.67%)	
Cardiovascular and cerebrovascular diseases	14 (17.5%)		
Malignant tumors	10 (12.50%)	3 (20%)	
Severe pancreatitis	6 (7.50%)	2 (13.33%)	
Severe trauma	6 (7.50%)	3 (20%)	
Severe hemorrhagic	4 (5%)		
APACHEⅡscore	28 ± 9.25	33 (24–35)	0.57
Site of infection			
Lung	65 (81.25%)	11 (71.33%)	
Bloodstream	7 (8.75%)	3 (20%)	
Abdominal	8 (10%)	2 (13.33%)	
Intracranial	3 (3.75%)		
Urinary tract	1 (1.25%)		
Skin and soft tissue	3 (3.75%)		
PMB treatment			
PMB loading dose (mg/kg)	78 (97.50%)	12 (80%)	
PMB maintenance dose (mg/kg)	1.31 ± 0.25	1.08 ± 0.07	0.72
PMB treatment duration (days)	13 (9–16)	12 (9–15)	0.31
Nebulization	51 (63.75%)	9 (60%)	
C_trough, ss_ (mg/L)	1.69 (0.78–3.29)	1.88 (1.12–3.19)	0.44
C_peak, ss_ (mg/L)	5.73 (3.96–7.64)	4.34 (3.48–6.00)	0.25
Equation-based AUC_ss,24h_ (mg·h/L)	102.12 ± 57.86	90.5 ± 43.44	0.67
Model-based AUC_ss,24h_ (mg·h/L)	74.07 (55.81–94.07)	67.67 (55.81–94.07)	0.35
Co-administered antimicrobial drugs			
β-lactam antibiotics	66 (82.5%)	11 (73.33%)	
Glycopeptide antibiotics	15 (18.75%)	4 (26.67%)	
Tigecycline	10 (12.50%)	1 (6.67%)	
Quinolone	3 (3.75%)		
Omadacycline	5 (6.25%)	3 (20%)	
Amikacin	3 (3.75%)		
Laboratory parameters			
BUN (mmol/L)	12.46 (8.59–22.27)	16.79 ± 2.42	0.34
Creatinine (μmol/L)	79.70 (51.60–158.90)	87.50 (51.80–116.50)	0.96
Creatinine clearance (mL/min)	75.99 (38.46–130.58)	60.56 (35.41–131.65)	0.75
eGFR (80–120 mL/min/1.73m^2^)	80.87 (39.40–133.47)	70.60 (45.99–141.74)	0.92
ALT (IU/L)	32.20 (18.50–56.65)	31.10 (24.60–95.60)	0.38
AST (IU/L)	46.30 (31.15–74.27)	67.50 (35.40–84.50)	0.45
TP (g/L)	60.59 ± 7.1	60.20 ± 2.41	0.20
ALB (g/L)	34.13 ± 4.2	33.48 ± 0.83	0.42
TBIL (μmol/L)	17.30 (11.40–36.60)	17.40 (11.60–25.08)	0.92
HGB	82 (74–89)	82.20 ± 4.50	0.33
WBC (10^9^/L)	9.57 (6.67–13.92)	13.36 ± 1.77	0.13
PLT (10^9^/L)	163.50 (84.5–266.25)	182 (120–424)	0.38
IL-6 (ng/L)	58.54 (24.16–141.40)	70.60 (47.83–371.30)	0.61
INR	1.16 (1.06–1.30)	1.19 ± 0.05	0.96
APTT (sec)	35.50 (29.90–41.70)	33 (29.30–37.87)	0.58
Fib	4.10 (2.40–5.10)	3.94 ± 0.47	0.79

Abbreviations: AUC_ss, 24h,_Concentration-time curve at steady state over 24 h; APACHE Ⅱ, Acute Physiology and Chronic Health EvaluationⅡ; C_trough_, steady-state trough concentration; C_peak_, steady-state peak concentration; BUN, blood urea nitrogen; eGFR, estimated glomerular filtration rate; ALT, Alanine Aminotransferase;AST, aspartate aminotransferase; TP, total protein; ALB, albumin; TBIL, total bilirubin; HGB, hemoglobin; WBC, white blood cell; PLT, platelet count;IL-6, interleukin; INR, international normalized ratio; APTT, activated partial thromboplastin time; Fib, fibrinogen.

The patients were divided into a modeling group and a validation group based on the chronological order of enrollment and in accordance with the inclusion and exclusion criteria. External validation of the final model was performed using the validation group data. The mean prediction error (MPE), mean absolute prediction error (MAPE) (Equations 1, 2), F_20_ (20% of the absolute value of the prediction error), and F_30_ were calculated by comparing the predicted values with the observed values. Model performance was considered acceptable if MPE% ≤ ±20%, MAPE% ≤ 30%, F_20_ ≥ 35%, and F_30_ ≥ 50% (Mao et al., 2018).

$$\text{MPE}\%=\frac{1}{N}\sum_{i=1}^{N}\left(\frac{{\text{pred}}_{i}-{\text{obs}}_{i}}{{\text{obs}}_{i}}\right)\times 100$$ (1)

$$\text{MAPE}\%=\frac{1}{N}\sum_{i=1}^{N}\left|\frac{{\text{pred}}_{i}-{\text{obs}}_{i}}{{\text{obs}}_{i}}\right|\times 100$$ (2)

pred_i_ denotes the i-th predicted value, and obs_i_ its corresponding observed value.

### Monte Carlo simulation

Using the final PopPK model, 1,000 simulations were conducted for commonly used maintenance doses in ICU patients, specifically 50 mg, 75 mg, 100 mg, and 125 mg every 12 h (q12h) with a 1-hour infusion time. According to clinical research (Tang et al., 2023), PMB has demonstrated better clinical efficacy in treating CRO caused nosocomial pneumonia when AUC_ss,24h_/minimum inhibitory concentration (MIC) ≥ 66.9. Given that the majority of patients in this study (65/80, 81.25%) had pulmonary infections, we selected AUC_ss,24h_/MIC ≥ 66.9 as the PK/PD target, with a 90% target attainment probability (PTA) was considered to be effective. Furthermore, to mitigate the potential risk of nephrotoxicity associated with excessively high AUC_ss,24h_, we defined the therapeutic window for AUC_ss,24h_ as 50–100 mg·h·L^-1^ (Tsuji et al., 2019; Yang et al., 2022). We analyzed the probability of the simulated population achieving this therapeutic window.

### Data analysis

Data analysis was performed SPSS (version 26.0, IBM, United States) and GraphPad Prism (version 8.3.0, CA, United States). Variables with a normal distribution were expressed as mean ± standard deviation (SD), while non-normally distributed variables were reported as median (interquartile range, IQR). Categorical data were presented as percentages (%). Comparisons between two groups were conducted using the Mann-Whitney U test for non-normally distributed data and the independent t-test for normally distributed data. The estimation of AUC_ss,24h_ used the two-point method was presented in Equations 3–5 (Liu et al., 2023), while the estimation of AUC_ss,24h_ based on the PopPK model was obtained by dividing the total 24-hour drug dose by the individual clearance. Bland-Altman analysis was performed to evaluate the consistency of AUC_ss,24h_ estimates obtained by the two methods. AUC_ss,24h_ values were categorized as “within the therapeutic window” (50–100 mg·h·L^-1^), and McNemar’s test and Kappa test were applied to assess the consistency of AUC_ss,24h_ classifications.

$$k_{e}=\frac{\ln C_{\text{peak},\text{ss}}-\ln C_{\text{trough},\text{ss}}}{\tau -\text{Infusion}}$$ (3)

$$C_{\text{soi}}^{\prime }\;\;=\frac{C_{\text{peak},\text{ss}}}{e^{-k_{e}\cdot \text{Infusion}}}$$ (4)

$${\text{AUC}}_{\text{ss},24h}=\frac{{C_{\text{soi}}}^{\prime }-C_{\text{trough},\text{ss}}}{k_{e}}\times n$$ (5)

Infusion time; τ: dosing interval; Csoi' is the exploratory concentration at the start of dosing based on the one-compartment linear elimination pharmacokinetic assumption; k_e_: elimination rate constant; n is the number of doses within 24 h.

## Results

### Patient characteristics

The modeling group consisted of 80 patients with 184 PMB blood concentration samples, while the validation group included 15 patients with 30 samples. 11 patients in the modeling group underwent repeated sampling. Clinical characteristics and laboratory parameters are summarized in Table 1, showing no significant differences between the two groups. All patients were infected with CRO, in the modeling group, *carbapenem-resistant Acinetobacter baumannii* (CRAB) was the most common infection (70 cases), followed by *carbapenem-resistant Enterobacterales* (CRE; 56 cases, comprising 45 *Klebsiella pneumoniae*, 4 *Enterobacter cloacae*, 3 *Serratia marcescens*, 2 *Escherichia coli*, and 2 *Citrobacter freundii*), and *carbapenem-resistant Pseudomonas aeruginosa* (CRPA; 15 cases). In the validation group, CRE was more prevalent (11 cases) than CRAB (10 cases). In the modeling group, the MICs of PMB against CRO strains were: ≤0.5 mg·L^-1^ in 72.25% (57/80), 1 mg·L^-1^ in 13.75% (11/80), 2 mg L^-1^ in 10% (8/80), and 16 mg·L^-1^ in 3.75% (3/80). In the validation group, MIC values were ≤0.5 mg·L^-1^ in 60% (9/15), 1 mg·L^-1^ in 26.67% (4/15), and 2 mg·L^-1^ in 13.33% (2/15), with no statistically significant difference observed (P = 0.21).

### PopPK model analysis and validation

A one-compartment model with first-order elimination best fit the population data of PMB in critically ill patients. Inter-individual variability was described using an exponential random effects model, while residual variability was described using both proportional and additive error models. Among the covariates evaluated, CrCL and platelet count (PLT) count were found to significantly influence CL, whereas no covariates had a significant effect on V_d_ (volume of distribution). The final PK model Equations 6, 7 is as follows:

$$\text{CL}=2.03\times {\left(\text{CrCL}/{\text{CrCL}}_{\text{median}}\right)}^{0.26}\times {\left(\text{PLT}/{\text{PLT}}_{\text{median}}\right)}^{\left(-0.14\right)}\times \exp\left(\eta \text{CL}\right)$$ (6)

$$V=18L\times \exp\left(\eta_{v}\right)$$ (7)

**FIGURE 1:**
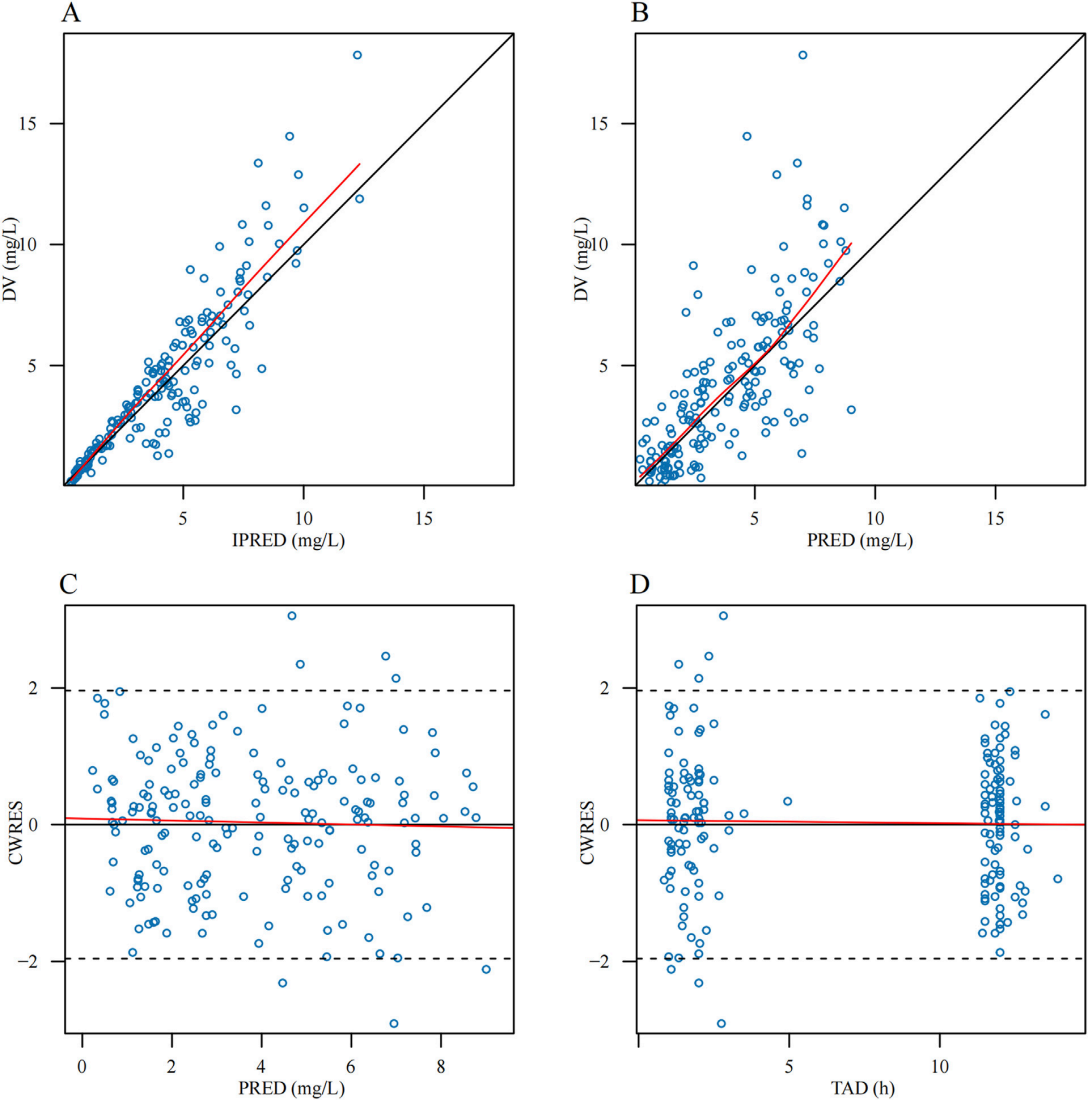
Goodness-of-fit plots of the final population pharmacokinetic model for polymyxin B. **(A)** Observed concentration (DV) versus individual prediction (IPRED) **(B)** DV versus population prediction (PRED) **(C)** Conditional weighted residuals (CWRES) versus PRED **(D)** CWRES versus time after dose (TAD). The red lines represent the locally weighted scatter plot smoothing (LOESS) curves.

**FIGURE 2:**
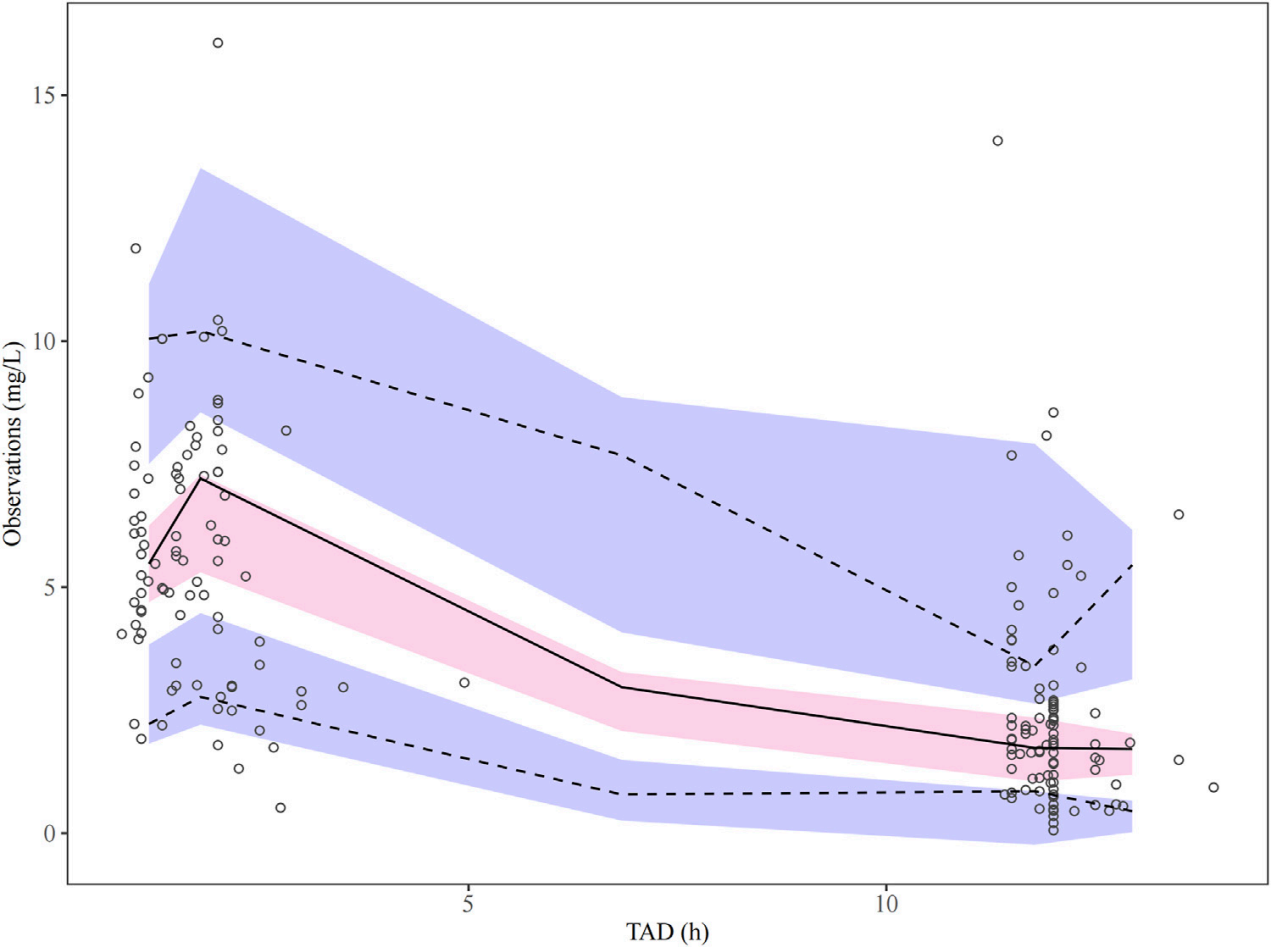
Visual predictive check plots for the PopPK final model. The solid black line indicates the median of the observed data, while the dashed lines show the 5th and 95th percentiles. The shaded regions represent the 95% confidence intervals for the 5th, 50th, and 95th percentiles derived from the simulations.

**FIGURE 3:**
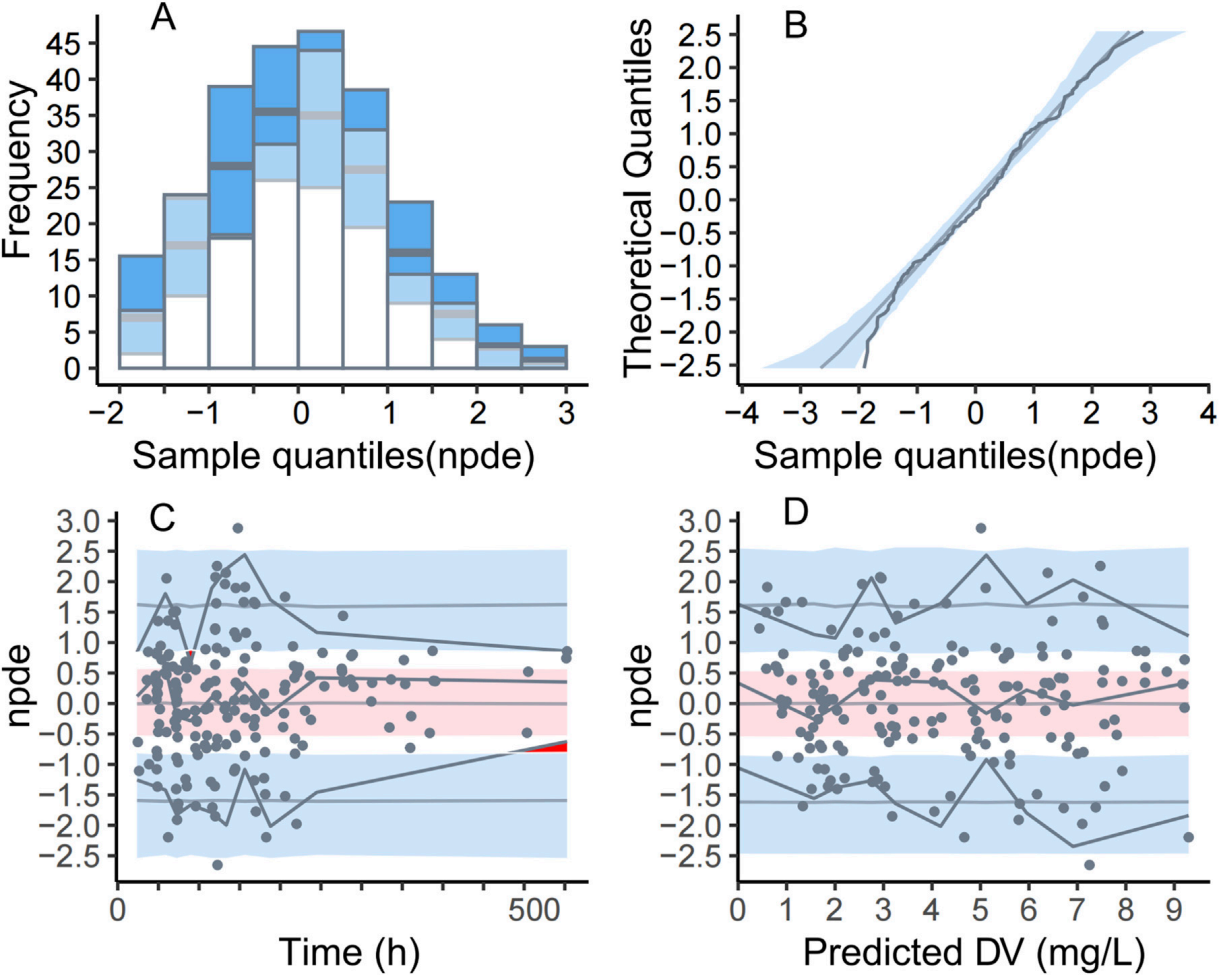
Normalized Prediction Distribution Errors (NPDE) plot for the final PopPK model. **(A)** Histogram of NPDE overlaid with the density plot of a standard normal distribution **(B)**. Q-Q plot of NPDE against the standard normal distribution **(C)**. Scatter plot of NPDE versus time after the first dose **(D)**. Scatter plot of NPDE versus population predicted (PRED) The dots represent the NPDE calculated from the dataset. In panel B, the blue shaded area represents the 95% prediction interval for a standard normal random variable. In panels C and D, the red shaded area corresponds to the prediction interval for the median (50^th^ percentile) of the NPDE, while the blue shaded area represents the prediction interval for the 5^th^ and 95^th^ percentiles. The solid lines depict the evolution of observed data across percentiles compared to model predictions.

Parameter estimates and GOF plots for the base model were presented in Supplementary Table S1 and Supplementary Figure S1 (Supplementary Material). The GOF plot (Figure 1) of the final model demonstrated strong agreement between observed and predicted values. The bootstrap median (Table 2) closely matched the population estimates of the final model, further confirming the robustness of the population PK model. The observed-versus-predicted plot showed a random scatter, indicating no systematic bias and suggesting that the model accurately describes the concentration data. The VPC plot (Figure 2) demonstrated that the median of the model predictions closely aligns with the median of the observed data, and most observed data points fall within the model’s 95% prediction interval. This indicated that the model has strong predictive ability and reasonable variability. Statistical tests for NPDE results included: t-test (P = 1), Fisher variance test (P = 1), Shapiro-Wilk normality test (P = 0.30), and Global test (P = 0.30). The NPDE histogram and Q-Q plot (Figure 3) showed that prediction errors were close to zero and symmetrically distributed, conforming to the normality assumption. NPDE showed no significant variation over time or across predicted concentrations, indicating that the model’s predictive performance is consistent and stable across various time points and concentration levels. External validation, conducted with 15 patients using the final model, showed an MPE% of 2.69%, MAPE% of 28.45%, F_20_ of 36.67%, and F_30_ of 73.33%, confirming acceptable predictive performance.

**TABLE 2:** PopPK parameter estimates in the final model and bootstrap.

	Final model		Bootstrap	
Parameter	Estimate	RSE (%)	Median	95%CI
CL (L·h^-1^)	2.03	5.30	2.02	1.81 ∼ 2.24
V(L)	18	5.30	17.98	16.30 ∼ 19.80
dCLdCrCL	0.26	20.40	0.26	0.15 ∼ 0.36
dCLdPLT	−0.14	24.20	−0.14	−0.22 ∼ -0.066
Inter-individual variability				
ηCL (%)	38.50	10.70	38.03	29.70 ∼ 46.60
Residual variability				
Proportional error	0.30	8.20	0.30	0.25 ∼ 0.35
Additive error	0.21	28.40	0.21	0.070 ∼ 0.35

Abbreviations: CL, clearance; V, volume of distribution; RSE, relative standard error; CI, confidence interval; dCLdCrCL, fixed parameter coefficient of creatinine clearance (CrCL) to CL; dCLdPLT, fixed parameter coefficient of PLT, to CL; η, variance of inter-individual variability.

### Comparison of the two AUC_ss,24h_ estimation methods

**FIGURE 4:**
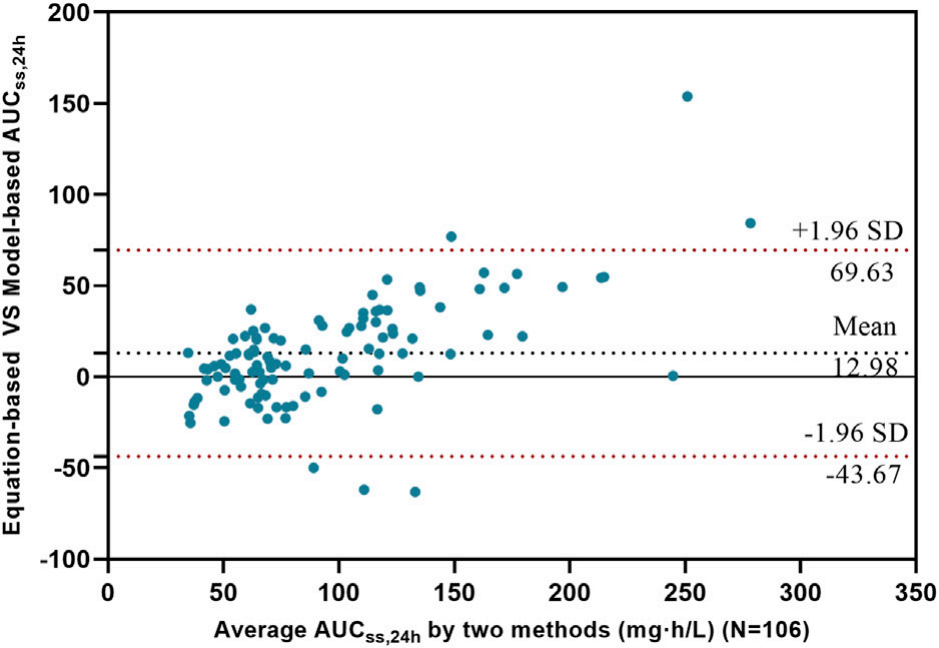
Bland-Altman analysis of AUC_ss,24h_ calculated by the two methods The black solid line represents the mean difference of AUC_ss,24h_ calculated by the two methods, while the red dashed lines represent the 95% confidence interval of the differences.

A total of 106 AUC_ss,24h_ values were obtained from the modeling group (91 values) and the validation group (15 values). The AUC_ss,24h_ estimated by the PPK model within the range of 50–100 was 51.89% (55/106), while the AUC_ss,24h_ estimated using the two-point method was 46.23% (49/106). The Bland-Altman analysis results were shown in Figure 4. The mean difference of AUC_ss,24h_ between the two methods was 12.98 mg·h·L^-1^, indicated a small average difference between the methods. The majority of the sample differences fell within the 95% confidence interval, demonstrated consistency between the two estimation methods in most samples. The Kappa test revealed moderate agreement between the two methods (κ = 0.51, P < 0.001), while McNemar’s test showed no significant difference in classification outcomes (P = 0.33), indicating overall consistency with minor discrepancies.

### Monte Carlo simulation

**FIGURE 5:**
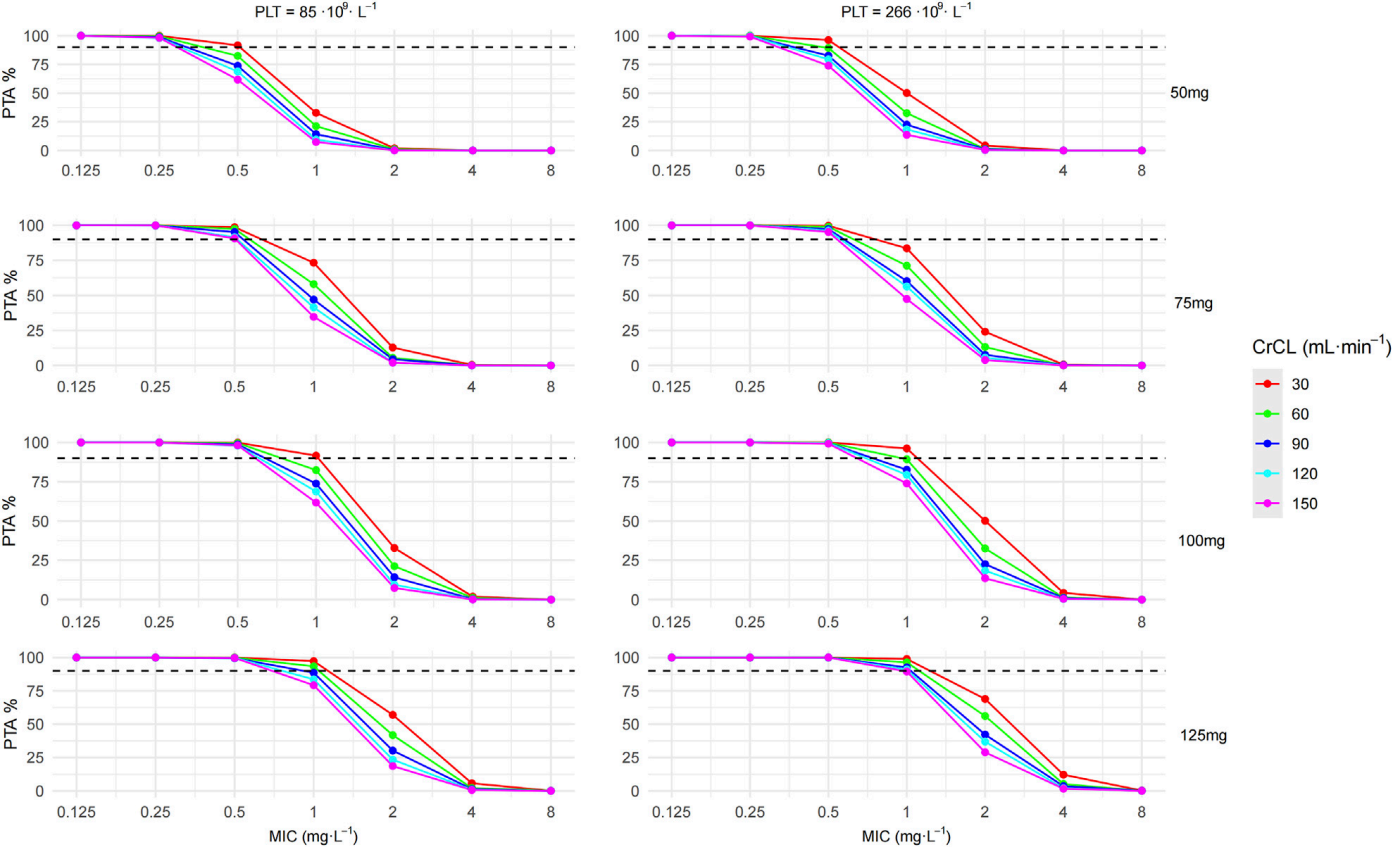
Probability of target attainment (PTA) for polymyxin B at a PK/PD target of AUC_ss,24h_/MIC ≥ 66.9.

To evaluate the impact of varying renal function levels, CrCL values of 30, 60, 90, 120, and 150 mL. min^-1^ were used in the simulation. Since PLT levels in the modeling data are concentrated in the low and normal PLT ranges, the simulation used PLT at the quartiles of the modeling data: 85·10^9^·L^-1^ and 266·10^9^·L^-1^. Simulation results were presented in Figure 5 and Table 3. Forty different clinical scenarios were simulated. At MIC ≤ 0.5 mg·L^-1^, PTA ≥ 90% was achieved in all groups except the 50 mg q12h maintenance dose group. PTA decreased as CrCL increased for the same maintenance dose and PLT level. Compared with maintenance dose and CrCL levels, PLT levels seem to exert a relatively minor influence on the PTA and proportion of AUC_ss,24h_ within the therapeutic window. In the 50 mg q12h group, the proportion of AUC_ss,24h_ within the therapeutic window decreased as CrCL increased, whereas an opposite trend was noted in the 100 mg and 125 mg groups. Overall, the 75 mg and 100 mg q12h groups had a slightly higher (75 mg 58.13%; 100 mg 46.78%; 50 mg 42.40%; 125 mg 14.66%) proportion of AUC_ss,24h_ within the therapeutic window compared to other groups.

**TABLE 3:** The AUC_ss,24h_ from simulated populations at different CrCL and PLT count.

Dose (mg)	CrCL(mL·min^-1^)	PLT = 85·10^9^·L^-1^			PLT = 266·10^9^·L^-1^		
		AUC_ss,24h_ < 50 mg·h·L^-1^ (%)	AUC_ss,24h_ = 50–100 mg·h·L^-1^ (%)	AUC_ss,24h_ > 100 mg·h·L^-1^ (%)	AUC_ss,24h_ < 50 mg·h·L^-1^ (%)	AUC_ss,24h_ = 50–100 mg·h·L^-1^ (%)	AUC_ss,24h_ > 100 mg·h·L^-1^ (%)
50	30	35.2	58	6.8	24.3	59.4	16.3
	60	51.8	45	3.2	37.2	55.8	7
	90	64.4	33.8	1.8	50.2	45.2	4.6
	120	69.9	29	1.1	55.5	41.2	3.3
	150	76.4	22.7	0.9	63.8	34	2.2
75	30	8.3	58.9	32.8	3.7	46.2	50.1
	60	17.4	61.4	21.2	10.5	57	32.5
	90	25.8	60	14.2	16.8	60.7	22.5
	120	30.6	59.9	9.5	20.5	61.1	18.4
	150	37.3	55.3	7.4	25.6	60.8	13.6
100	30	2.2	33.8	64	0.6	24.2	75.2
	60	4.4	48	47.6	2.3	35.5	62.2
	90	8.4	56.7	34.9	4.7	46	49.3
	120	13.4	56.7	29.9	6.2	50	43.8
	150	15.6	61	23.4	8.4	55.9	35.7
125	30	0.5	17	82.5	0.1	10.4	89.5
	60	1.4	29.2	69.4	0.4	20.6	79
	90	3.2	38.2	58.6	1.4	28.8	69.8
	120	4.8	42	53.2	2	32.8	65.2
	150	4.9	50.6	44.5	2.6	39.2	58.2

Abbreviations: AUC_ss, 24h_, an area under the plasma concentration time curve across 24 h at steady state; PLT, platelet count; CrCL, creatinine clearance.

## Discussion

In this study, we develop a PopPK model for critically ill patients receiving PMB therapy using a two-point method based on C_trough,ss_ and C_peak,ss_. Furthermore, it is the first to compare AUC_ss,24h_ estimates from the first-order elimination equation method based on C_trough,ss_ and C_peak,ss_ with those estimated using a PopPK model.

Our findings revealed that the one-compartment model with first-order elimination adequately described the population data, with CrCL and PLT identified as covariates influencing PMB PK. This model was found to be suitable for application in critically ill patients. Model-based simulations suggested that PLT levels had a minimal impact on the PTA. At MIC ≤ 0.5 mg·L^-1^, commonly used maintenance doses in critically ill patients (50, 75, 100, 125 mg q12h) generally achieved 90% PTA, except in the 50 mg maintenance dose group with high CrCL. The 75 mg and 100 mg q12h regimens showed a higher proportion of AUC_ss,24h_ values within the therapeutic window compared to other regimens, suggesting these dosing strategies may be optimal for this population. Additionally, CrCL levels should be considered for further optimization of individualized dosing. The comparison of AUC_ss,24h_ estimation methods showed good consistency, supporting the use of the C_trough,ss_ and C_peak,ss_ two-point sampling strategy for routine TDM.

Compared to other one-compartment PopPK studies of PMB (Table 4) (Kubin et al., 2018; Manchandani et al., 2018; Yu et al., 2020; Crass et al., 2021; Li et al., 2021), our results were similar to those of Manchandani et al. (CL = 2.5 L·h^-1^) and Kubin et al. (CL = 2.37 L·h^-1^) in CRO-infected patients, highlighting the significant influence of renal function on CL. In contrast, the lower CL observed in previous studies by Li et al. (1.18 L·h^-1^) and Yu et al. (1.59 L·h^-1^) may reflect differences in renal function and patient characteristics. Our study reported a value of 18 L, which is lower than the values reported in earlier studies by Manchandani et al. (34.3 L) and Kubin et al. (34.40 L), but is more closely aligned with the values from Li et al. (12.90 L) and Crass et al. (12.70 L). Variations in V_d_ may reflect differences in fluid status and organ function, as critically ill patients often experience tissue edema and hemodynamic instability, which affect drug distribution and explaining discrepancies across studies.

**TABLE 4:** Pharmacokinetics of polymyxin B obtained from different studies.

Study	Manchandani P et al.	Li et al.	Yu et al.	Kubin CJ et al.	Crass RL et al.
Population	CRO-infected patients	Kidney transplant patients	Critically ill patients	CRO-infected patients	Cystic fibrosis patients
Covariate	TBW	CrCL	CrCL	-	TBW
CL (L·h^-1^)	2.50	1.18	1.59	2.37	2.09
Vd(L)	34.30	12.90	20.50	34.40	12.70

Abbreviations: TWB, total body weight; CrCL, creatinine clearance.

The association between PMB pharmacokinetics and CrCL is still debated. A study (Sandri et al., 2013b) reported that the urinary recovery of PMB in 17 patients ranged from 0.98% to 17.40%, while another study (Yu et al., 2020) reported a recovery rate of 23.56% in four patients. This suggested significant interindividual variability in PMB renal clearance. Our findings indicated that CrCL is a significant covariate influencing PMB pharmacokinetics, consistent with previous PopPK studies (Avedissian et al., 2018; Wang et al., 2020; Yu et al., 2020; Li et al., 2021; Wang et al., 2021; Luo et al., 2022; Ye et al., 2022). In this study, the similar proportions of patients with and without renal impairment represented a wide range of renal function levels. Simulations indicated higher PTA in patients with renal impairment. This may result from impaired glomerular filtration, leading to decreased PMB clearance and increased drug exposure. With increased maintenance doses, patients with renal impairment exhibited higher drug exposure, leading to more AUC_ss,24h_ values exceeding 100 mg·h·L^-1^.

In critically ill patients, conditions such as inflammation and organ dysfunction, especially in sepsis, can alter PLT levels, potentially affecting drug metabolism and clearance (van Dalen and Vree, 1990; van der Poll et al., 2013). Hypoalbuminemia, frequently observed in critically ill patients, may correlate with changes in PLT, influencing drug plasma protein binding and concentrations (Roberts et al., 2014b).Renal function changes, such as acute kidney injury, may also be associated with variations in PLT, thereby influenced drug clearance (Ulldemolins et al., 2011; Chawla et al., 2014). Our study identified PLT as a significant factor influencing the pharmacokinetics of PMB, and to our knowledge, this is the first report of such a finding. This finding opens new avenues for pharmacokinetic modeling and underscores the need for further investigation into the relationship between PLT and PMB pharmacokinetics.

Our study shows that although there is a small bias between the two AUC_ss,24h_ estimation methods, this difference is clinically acceptable and provides a simple yet reliable tool for clinical TDM without the need for complex modeling. However, the development of individualized dosing regimens still relies on the PopPK model, which integrates patient characteristics to enable precise predictions and adjustments, thereby optimizing the efficacy and safety of PMB therapy.

Despite the significant findings, our study has some limitations. First, we did not correlate PMB PK/PD parameters with patient outcomes, so the relationship between PMB exposure and clinical outcomes remains unclear. Second, most patients in our study had PLT counts within or below normal ranges, and to minimize simulation errors, we excluded populations with elevated PLT levels from modeling. Consequently, our findings may not apply to populations with elevated PLT counts. This novel observation should be interpreted with caution, especially as prior studies have not reported PLT’s influence on PMB pharmacokinetics. Lastly, we measured total plasma concentrations of PMB without evaluating free drug levels. If significant variability in albumin levels exists among patients, the applicability of our findings could be limited.

## Conclusion

This study established a PopPK model for PMB in critically ill patients, identifying CrCL and PLT as covariates influencing PMB clearance. The 75 mg q12h and 100 mg q12h dosing regimens seem appropriate for critically ill patients; however, CrCL levels should be considered when selecting between these regimens to guide individualized dosing. The two-point sampling strategy based on C_trough,ss_ and C_peak,ss_ can be applied for routine TDM of PMB.

## Data Availability

The raw data supporting the conclusions of this article will be made available by the authors, without undue reservation.

## Abbreviations

ALB, Albumin; IL-6, Interleukin-6; ALT, Alanine Aminotransferase; INR, International normalized ratio; APACHE, Ⅱ Acute Physiology and Chronic Health Evaluation Ⅱ; IPRED, Individual prediction; APTT, Activated partial thromboplastin time; MIC, Minimum Inhibitory Concentration; AST, Aspartate Aminotransferase; NPDE, Normalized prediction distribution error; AUC_ss,24h_, Concentration-time curve at steady state over 24 h; OFV, Objective Function Value; BUN, Blood urea nitrogen; PK/PD, pharmacokinetics/pharmacodynamics; CI, Confidence Interval; PLT, Platelet count; CL, Systemic Clearance; PMB, Polymyxin B; C_peak,ss_, Steady-state peak concentration; PopPK, Population Pharmacokinetics; CRAB, carbapenem-resistant Acinetobacter baumannii; PRDE, Population prediction; CrCL, Creatinine Clearance; PTA, Probability of Target Attainment; CRE, carbapenem-resistant Enterobacterales; TBIL, Total bilirubin; CRO, Carbapenem-resistant organisms; TBW, Total body weight; CRKP, Carbapenem-resistant Klebsiella pneumoniae; TDM, Therapeutic Drug Monitoring; C_trough,ss_, Steady-state trough concentration; TP, Total protein; CWRES, Conditional weighted residuals; UPLC-MS/MS, Ultra-performance liquid chromatography-tandem mass spectrometry; DV, Observed concentration; Vd, Volume of Distribution of the Central Compartment; eGFR, Estimated glomerular filtration rate; VPC, Visual predictive check; Fib, Fibrinogen; WBC, White Blood Cell; HGB, Hemoglobin.
