# Supplementary figures and images for: Population pharmacokinetics of polymyxin B in critically ill patients with carbapenem-resistant organisms infections: insights from steady-state trough and peak plasma concentration
Source: Front Pharmacol. 2025 Mar 12;16:1511088. doi: 10.3389/fphar.2025.1511088 (PMC11936910; doi:10.3389/fphar.2025.1511088)

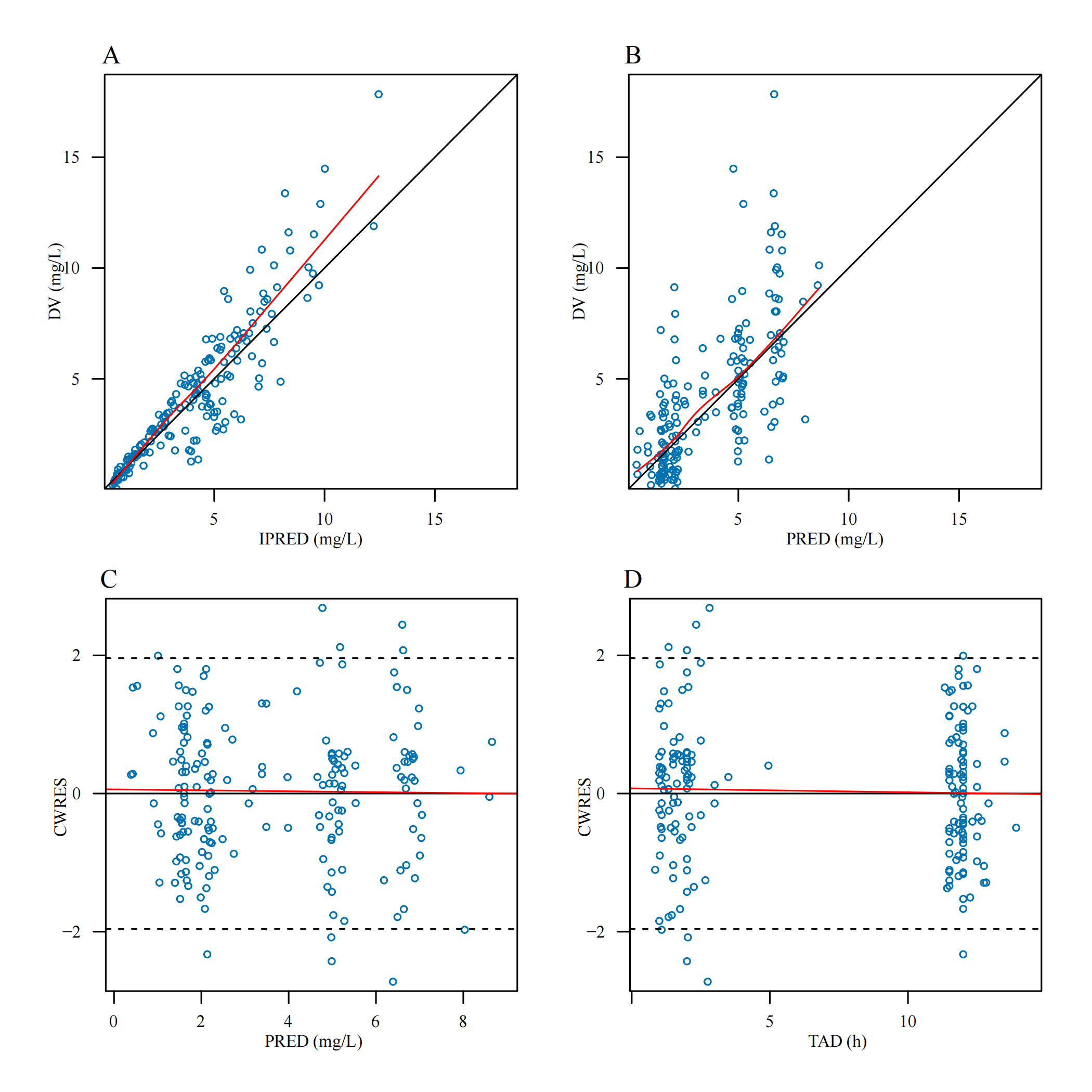

Supplement: Supplementary file 1 [file Image1.tif]
